# For a Burn

**Published:** 1891-11

**Authors:** 


### FOR A BURN.

If a person has been burned by the clothes catching fire, remove the clothing as soon as possible, taking care to keep the burned surface drenched with tepid water ; and be sure not to drag upon the injured skin in such a way as to pull it off, as it is the best possible protection for the tender flesh beneath.

When the clothing has been removed, keep the burned surface covered with cloths wrung out of soda water, made by dissolving a teaspoonful of soda in a pint of water. This is an effectual method of treating burns, and is far superior to the old dressing of carron oil, a mixture of linseed oil and lime water.

